# Short-term results of laparoscopic anti-reflux surgery with the RefluxStop device in patients with gastro-esophageal reflux disease and ineffective esophageal motility

**DOI:** 10.1007/s00423-024-03264-5

**Published:** 2024-02-29

**Authors:** Yannick Fringeli, Ioannis Linas, Ulf Kessler, Joerg Zehetner

**Affiliations:** 1 Department of Visceral Surgery, Hirslanden Clinic Beau-Site, 3013 Bern, Switzerland; 2 Department of Gastroenterology, Hirslanden Clinic Beau-Site, 3013 Bern, Switzerland

**Keywords:** Gastro-esophageal reflux disease, Ineffective esophageal motility, Esophageal dysmotility, Hiatal hernia, Anti-reflux surgery, RefluxStop

## Abstract

**Purpose:**

In gastro-esophageal reflux disease (GERD) requiring surgical treatment, concomitant ineffective esophageal motility (IEM) is a decisive factor in surgical planning, due to concern regarding dysphagia. Anti-reflux surgery with the RefluxStop device is a promising technique. We assessed initial feasibility and clinical outcomes of RefluxStop surgery in patients with GERD and IEM.

**Methods:**

Retrospective analysis of patients with GERD, hiatal hernia (HH), and IEM, who underwent surgery with RefluxStop at our institution and achieved 12-month follow-up. Technique feasibility was assessed, in addition to symptom resolution (GERD-HRQL questionnaire), adverse events, HH recurrence, dysphagia, and patient satisfaction. Placement of the device was confirmed by video fluoroscopy on postoperative day 1, and at 3 and 12 months.

**Results:**

Between June 2020 and November 2022, 20 patients with IEM underwent surgery with RefluxStop and completed 12-month follow-up. All patients reported typical symptoms of GERD, and 12 had preoperative dysphagia. The median HH length was 4.5 cm (IQR, 3.75–5). The median operating time was 59.5 min (IQR, 50.25–64) with no implant-related intra- or postoperative complications. No HH recurrence was observed. One patient reported persistent left-sided thoracic pain at 11 months post-surgery, which required diagnostic laparoscopy and adhesiolysis. Three patients reported severe postoperative dysphagia: balloon dilatation was performed towards resolution. The mean GERD-HRQL scores improved (from 40.7 at baseline to 4.8 at 3 months and 5.7 at 12 months (*p* <0.001)).

**Conclusion:**

RefluxStop surgery was feasible and offered effective treatment for this group of patients with GERD and IEM. All patients had complete resolution or significant improvement of GERD symptoms, and 90% of them were satisfied with their quality of life 1 year after surgery.

## Introduction

In the surgical treatment of gastro-esophageal reflux disease (GERD), laparoscopic hiatal hernia (HH) repair combined with fundoplication (complete or partial) is regarded as the standard of care. Other techniques, both laparoscopic and endoscopic, are constantly being developed, each with strengths and limitations such as degree of invasiveness, possibility of HH repair, and particular relative contraindications [1, 2]. In recent years, the RefluxStop device and its corresponding procedure have been introduced, with promising postoperative outcomes from the initial patient group published up to 1 year [3].

**Fig. 1:**
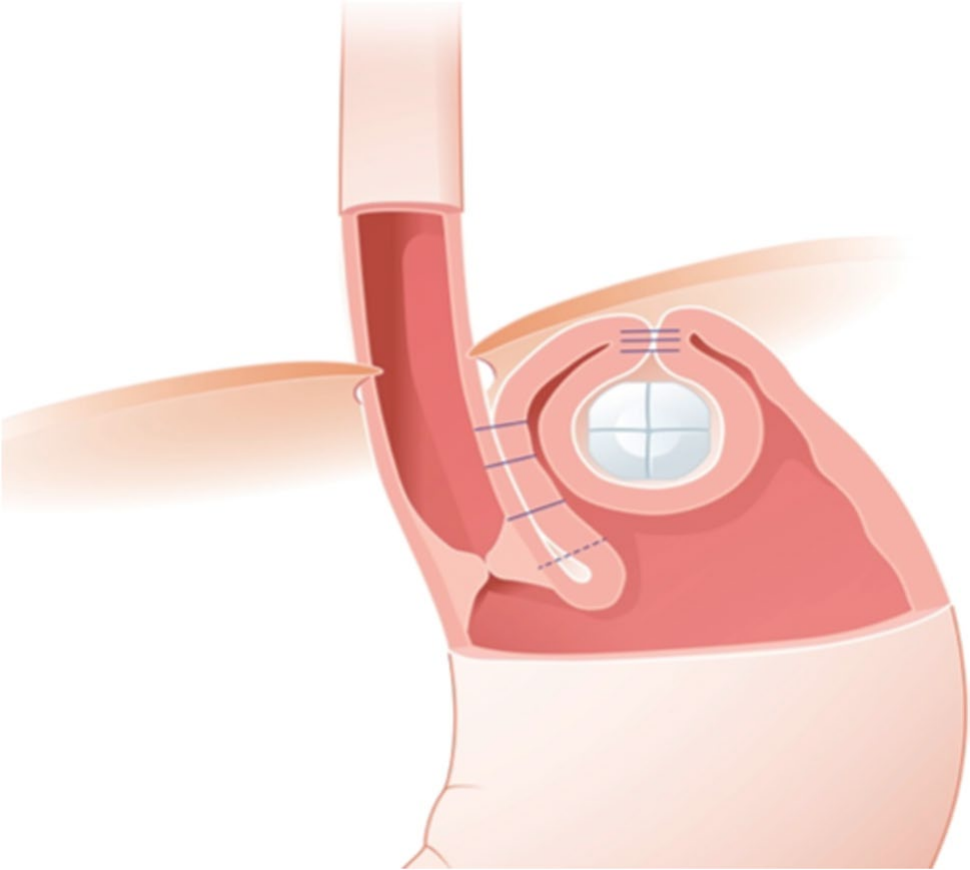
RefluxStop device in situ, showing the esophago-gastric plication and the implant in its fundic pouch. The implant is composed of five small parts bound together by an absorbable suture. Image used with permission from manufacturer

The concept of RefluxStop surgery is that, in addition to the HH repair performed during the procedure, a high mediastinal dissection, narrow esophago-gastric plication, and implantation of the nonactive device recreate an acute angle of His and ensure sufficient esophageal length within the abdomen, with the corresponding intra-abdominal pressure helping the lower esophageal sphincter to function correctly (Fig. 1) [3].

A particular claim of this technique is that, as the device sits in a fundic pocket adjacent to the esophagus (Fig. 1) rather than encircling it, and the plication is narrow (90–110°), it does not predispose to the dysphagia seen in other surgical repair techniques.

In clinical practice, the decision on the type of surgery performed should be made on an individual basis, taking into account not only the surgeon’s skillset and experience, but also the patient’s characteristics, such as predominant symptoms, hernia size, and presence of esophageal motility disorder (EMD). In the group of patients with preexisting ineffective esophageal motility (IEM), there are particular challenges to consider.

Ineffective esophageal motility comprises a heterogenous, minor motility disorder subgroup of EMDs [4]. Although EMDs may not always cause symptoms, they often coexist with GERD, IEM being the main EMD in GERD patients [5] with an overall incidence of 49.4% and prevalence of 20–30% [6]. Ineffective esophageal motility impairs esophageal clearance and contributes to GERD pathophysiology [4]. The current management of IEM relates to treatment of symptoms and concurrent reflux, if present [4].

In most centers that provide anti-reflux surgery, preoperative workup includes assessment of esophageal motility. Nissen fundoplication (360°) has generally been considered preferable for patients with normal esophageal motility, and partial fundoplication preferred in patients with ineffective or poor esophageal motility, due to concern regarding postoperative dysphagia risk. Among the more recent laparoscopic and endoscopic alternatives, magnetic sphincter augmentation (LINX™) is contraindicated in patients with preexisting IEM, since their motility is too weak to overcome the resistance of the device, leading to dysphagia [4, 7, 8].

Therefore, there is an unmet need in the treatment of this patient group. This is a particular area in which RefluxStop and its related procedure appear to be especially suitable, in that it does not encircle the esophagus and as such claims to reduce the risk of postoperative dysphagia. RefluxStop surgery is offered at our institution as one of the laparoscopic anti-reflux surgery (LARS) options for patients with GERD and concurrent HH, and we postulated that it would be beneficial for patients with IEM. However, the original study of this device [3] excluded patients with motility disorders; therefore, there is little information on this patient group besides what is done in individual centers. We report here our early experience and assessment of the feasibility, safety, and clinical outcomes of RefluxStop surgery in this patient group.

## Methods

### Study design and patient population

A retrospective chart review was performed for the first cohort of patients with GERD, HH, and concurrent IEM who underwent LARS with the RefluxStop (Implantica, Zug, Switzerland) implant by a single surgeon (J.Z.) in a private hospital setting (Hirslanden Clinic Beau-Site, Bern, Switzerland). Surgery with RefluxStop was offered as a LARS option to these patients, and details of the procedure and the availability of limited data were provided to the patients prior to obtaining informed consent. Ethical approval for this study was obtained from the local ethics board of Canton Bern, Switzerland (Ethics Approval No. 2018-01827).

The study included patients aged ≥18 years with documented GERD, concurrent with IEM (identified on video-esophagram with inefficient or slow emptying of the esophagus or on manometry with less than 70% contractile waves or an amplitude of less than 30 mm Hg), who underwent laparoscopic HH repair with the RefluxStop device. Patients <18 years of age, with HH >10 cm, long-segment Barrett’s esophagus (BE), or a history of esophageal or gastric surgery were excluded. Foreign patients were also excluded due to unreliability of follow-up data.

### Preoperative assessments

Preoperative workup comprised a standardized history and physical examination, upper endoscopy with biopsies of the distal esophagus, and a standardized questionnaire for reflux disease (GERD-HRQL, 0–75 points) including an additional quality-of-life question (Question: How satisfied are you with your current quality of life related to GERD?; Possible responses: “satisfied,” “neutral,” or “dissatisfied”). As part of the preoperative workup, patients underwent video-esophagram under fluoroscopy in a standardized fashion, with swallows of contrast-enhanced liquid medium in upright and supine position according to a protocol [9]. If, on video-esophagram, the assessment of esophageal motility was inconclusive, high-resolution manometry was performed at a specialized reflux center in selected patients. Patients with a large HH (with typical symptoms like heartburn and regurgitation or night-time aspiration), BE, or reflux esophagitis grade C or D according to the Los Angeles classification [10] were not further subjected to pH studies; in the remaining patients, severity of GERD was assessed with pH testing in the form of 24-h pH-impedance study or a 48-h pH study with the Bravo capsule.

### Surgical techniques for implantation of the RefluxStop device

Surgery with the RefluxStop device aims to maintain the lower esophageal sphincter in an intra-abdominal position and recreate the angle of His, which is often flattened out by the hiatal hernia, while avoiding encircling the esophagus. Rather than the 270- or 360-degree wrap of existing fundoplication techniques, the attachment between the stomach and esophagus is in the region of 90 to 110°. The device is sutured into a pocket created on the gastric fundus, to stabilize the fundus and avoid re-herniation of the lower esophageal sphincter (LES) into the thoracic cavity. The device and its corresponding surgical procedure have been described in a previous publication [3]. Below, we provide a brief description of key areas.

#### The device

The RefluxStop device consists of five small parts made of medical grade silicone held together by an absorbable suture (Fig. 1). The device is delivered into the abdominal cavity using a purpose-made deployment tool (Implantica, Zug, Switzerland) and implanted into the pouch on the outside of the gastric fundus.

#### Surgical technique

The surgical preparation for surgery with RefluxStop is similar to other LARS, with pneumoperitoneum installed in the left upper quadrant using a Veress needle, and trocars in the typical LARS positions. With an Optiview trocar, a camera is introduced about 5–7 cm above the umbilicus, paramedian to the left. A 10-mm trocar is placed in the left upper quadrant and a 5-mm trocar in the right upper quadrant and the left flank. A Nathanson liver retractor, held by an iron intern, elevates the left lobe of the liver, via epigastric access.

##### Dissection and hiatal hernia closure

The pars flaccida is opened, the right crus identified, and the esophagus visualized. The anterior aspect of the esophagus is dissected with caution to preserve the anterior vagus nerve, and the top of the left crus is identified. The short gastric vessels are taken down, and the complete left crus is visualized and freed up from adhesions. An easy-flow drainage tube of 18 cm length is placed around the distal esophagus, to be used for retraction.

Mediastinal dissection of the distal esophagus is performed, preserving the vagal nerves. The hiatal hernia is reduced, and the hernia sac resected. With sufficient dissection, an intra-abdominal length of at least 4.5 cm should be achieved with only slight traction on the esophagus.

The hiatus is closed using 2 to 3 figure-of-eight sutures with Gore sutures (Gore Inc., Sedona, USA), avoiding compression of the esophagus. If there is an excessive fat pad at the angle of His, it is further resected.

##### Plication and pouch creation

An esophago-gastric plication, using two rows of sutures with V-loc (Medtronic Inc., Dublin, Ireland) non-resorbable 3-0, recreates the angle of His. The first row of sutures approximates the esophagus and the gastric fundus, starting at the angle of His and working caudocranially until about 4 cm of the distal esophagus and the fundus are joined. Slight tension on the esophagus during this plication creates a downward movement, a maximum of 1.5 cm, of the angle of His to allow the surgeon to reach higher up the esophagus. The second row of sutures is placed 1–1.5 cm anteriorly to the first suture row, taking care to avoid creating folds or kinks. Then a single Gore suture is placed to secure the fundus at the top end between the 2 suture lines.

The prepared RefluxStop device is then introduced using its deployment tool. Next to the suture line and parallel to the esophagus, at the top of the fundus, the device is gently placed without tension into a fundic pocket. The device is secured in position with one suture row from cranial to caudal, and a second suture row from caudal to cranial, taking care to avoid narrowing or kinking of the esophagus, and without tension on the easy-flow. The deployment tool and the easy-flow drainage tube are then removed.

### Postoperative assessment and follow-up

As for all other LARS procedures performed at our institution, the protocol included a minimum 2 postoperative overnight stays. On postoperative day 1, all patients underwent a video-esophagram. Follow-up visits with history and physical examination were mandatory at 4 weeks, 3 months, and 12 months after the procedure. Patients were asked to fill out a standardized reflux questionnaire (GERD-HRQL) preoperatively and at 3 months and 12 months. Results were defined as excellent if the GERD-HRQL scores were between 0 and 5; good for scores between 6 and 10; fair for scores between 11 and 15; and poor for scores >15 or if the patient required reoperation. Video-esophagram was also repeated at 3 months and 12 months post-surgery. Reflux symptoms and dysphagia were recorded at each office visit. Postoperative surgical complications within 90 days were documented according to the Clavien–Dindo classification [11].

### Statistical analysis

Continuous variables were expressed as mean ± standard deviation or median with interquartile range (IQR) when appropriate and categorical variables were expressed as percentages and frequencies. Comparison of continuous variables was carried out using the Mann–Whitney–Wilcoxon test, and categorical variables were compared using the chi-square or Fisher’s exact test, as applicable. Statistical analyses were performed using GraphPad Prism version 9.5.0, GraphPad Software, San Diego, CA, USA, with a significance level of 0.05.

## Results

### Baseline characteristics and demographic details

**Fig. 2:**
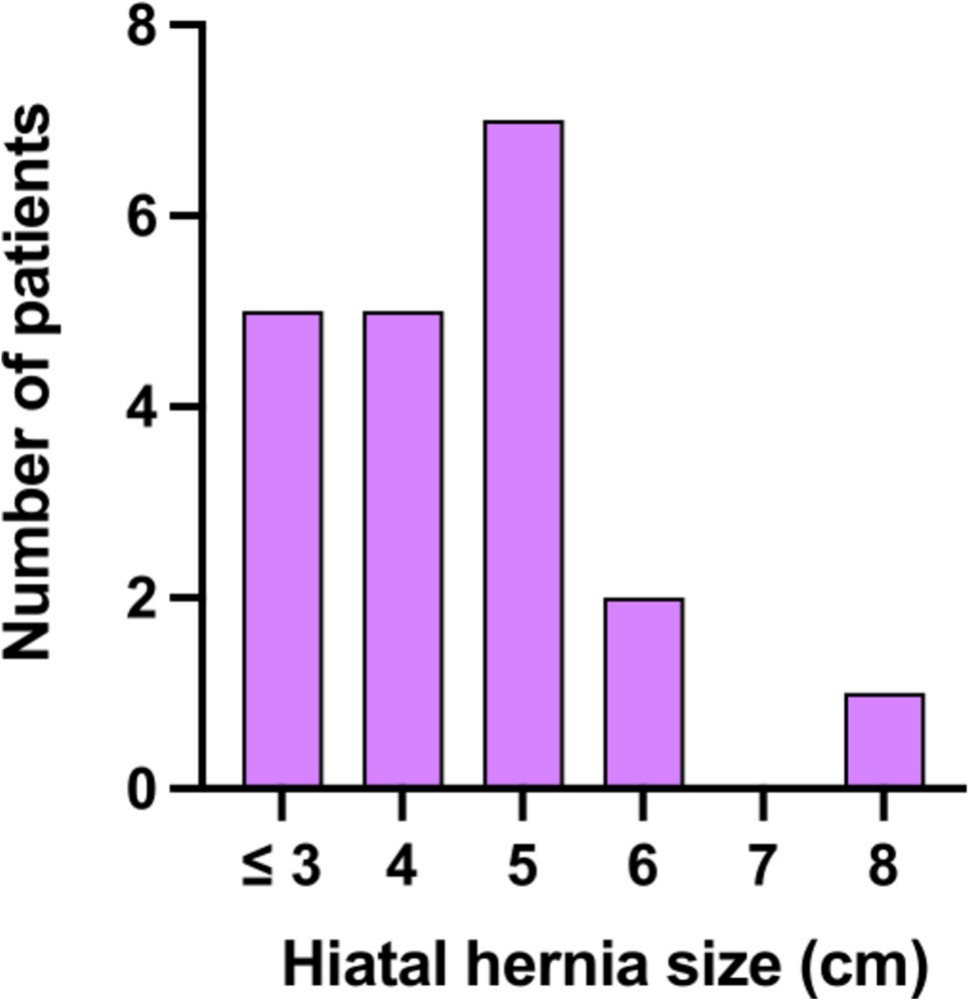
Distribution of the study population (*n*=20) according to the axial length of hiatal hernia (cm) before surgery

Among the first 60 patients who underwent LARS with the RefluxStop device, 20 were identified as having preoperative IEM and at least 12 months’ follow-up for analysis. The patients underwent surgery between June 2020 and November 2021, and 12 months’ follow-up was therefore achieved by November 2022. The demographics, baseline clinical parameters, and operative and postoperative characteristics of study participants are summarized in Table 1. Hiatal hernia axial length ranged from 3 to 8 cm (Fig. 2). All 20 patients had preoperative video-esophagram to evaluate esophageal motility, and high-resolution manometry was additionally performed in 6 patients, confirming IEM. Twelve patients (60%) had preoperative dysphagia. Complete video-esophagram follow-up (100%) was achieved in all patients at 3 and 12 months. All 20 patients provided responses to the standardized questionnaire (GERD-HRQL score) preoperatively and at 12 months, with 18 patients providing responses at the 3-month visit.

**Table 1:** Demographic, baseline clinical characteristics, and peri- and postoperative courses in patients with ineffective esophageal motility who underwent laparoscopic anti-reflux surgery with the RefluxStop implant

Parameter	*n* = 20
Demographics	
Sex (female)	10 (50%)
Age (years), mean (SD)	57.4 (12.6)
BMI (kg/m^2^), mean (SD)	26.5 (4.8)
ASA classification (1–6), median (IQR)	2 (2–3)
Reflux-related clinical parameters	
Axial length of hernia (cm), median (IQR)	4.5 (3.75–5)
Preoperative dysphagia	12 (60%)
Preoperative Barrett’s esophagus	6 (30%)
Preoperative reflux esophagitis grade C or D	4 (20%)
PPI use before surgery	20 (100%)
PPI use 12 months after surgery	4 (20%)
Perioperative characteristics	
Abdominal access	
Laparoscopic	19 (95%)
Conversion to open	1 (5%)
Reinforcement with Phasix-ST^®^ mesh	2 (10%)
Duration of operation (min), median (IQR)	59.5 (50.25–64)
Intraoperative complication	1 (5%)
Duration of hospital stay (days), median (IQR)	3.5 (3–4)
Postoperative characteristics	
Complications ≥ grade II within 90 days*	3 (15%)
Dysphagia requiring dilatation	3 (15%)
Reoperation within 12 months	1 (5%)
Recurrence of reflux within 12 months	1 (5%)
Migration of the implant within 12 months	1 (5%)

Values are *n* (%) unless stated otherwise. *BMI*, body mass index. *ASA*, American Society of Anesthesiologists. *PPI*, proton pump inhibitors

^*^Postoperative complications are graded according to the Clavien–Dindo classification system

### Perioperative and postoperative course

The RefluxStop procedure was feasible in all 20 patients. In 19 (95%) patients, the surgery was performed laparoscopically, and in one patient, who had had previous open surgery, the procedure was converted to open due to adhesions and bleeding while establishing laparoscopic access. Once the bleeding was controlled, the intended procedure was performed safely as described in the “Methods” section. The median operating time was 59.5 min (IQR, 50.25–64 min). In all patients, video-esophagram on day 1 confirmed adequate reduction of the HH and the correct location of the RefluxStop device. The median hospital stay was 3.5 days (IQR, 3–4 days). All patients tolerated a blended soft diet starting at day 1 for 7 days, followed by a soft diet for 3–4 weeks.

Three patients (15%) had persistent severe dysphagia requiring endoscopic dilatation after surgery. Two of these patients had suffered from dysphagia before surgery. The third patient had new-onset dysphagia and had received reinforcement of the hiatoplasty with a bioresorbable mesh (Phasix-ST^®^ mesh, Bard, BD, USA) due to large HH (5 cm) at the index operation. All three patients were successfully treated with repeated endoscopic dilatations (6, 4, and 3 sessions, respectively) with complete resolution of dysphagia thereafter.

One patient (5%) required reoperation during the 12-month follow-up period. A diagnostic laparoscopy with adhesiolysis was carried out 11 months after the initial operation due to persistent left-sided thoracic pain, which was considered unrelated to the device.

In one patient (5%), the 12-month video-esophagram showed that the implant had “migrated” from its initial position; that is, the five parts of the implant had exited the pocket along the deployment tool channel. The patient had no specific symptoms and scored “excellent” on the GERD-HRQL questionnaire (score of 0/75), as well as reporting complete satisfaction after surgery. On endoscopy, sufficient flap valve was seen, and the location of the implant was not detected. Computed tomography showed that 3 parts were sitting close to the stomach, while 2 parts were close to the spleen and greater omentum. With a diagnostic laparoscopy, all 5 parts were easily recovered. The reason for this was thought to be insufficient closure of the deployment tool channel.

No device-related re-operations were reported in the patient population. There were no reports of device-related complications at surgery, during the 12 months’ clinical follow-up, or on video-esophagram at day 1 and at 3- and 12-month follow-up.

### Clinical outcomes at 3 and 12 months

**Fig. 3:**
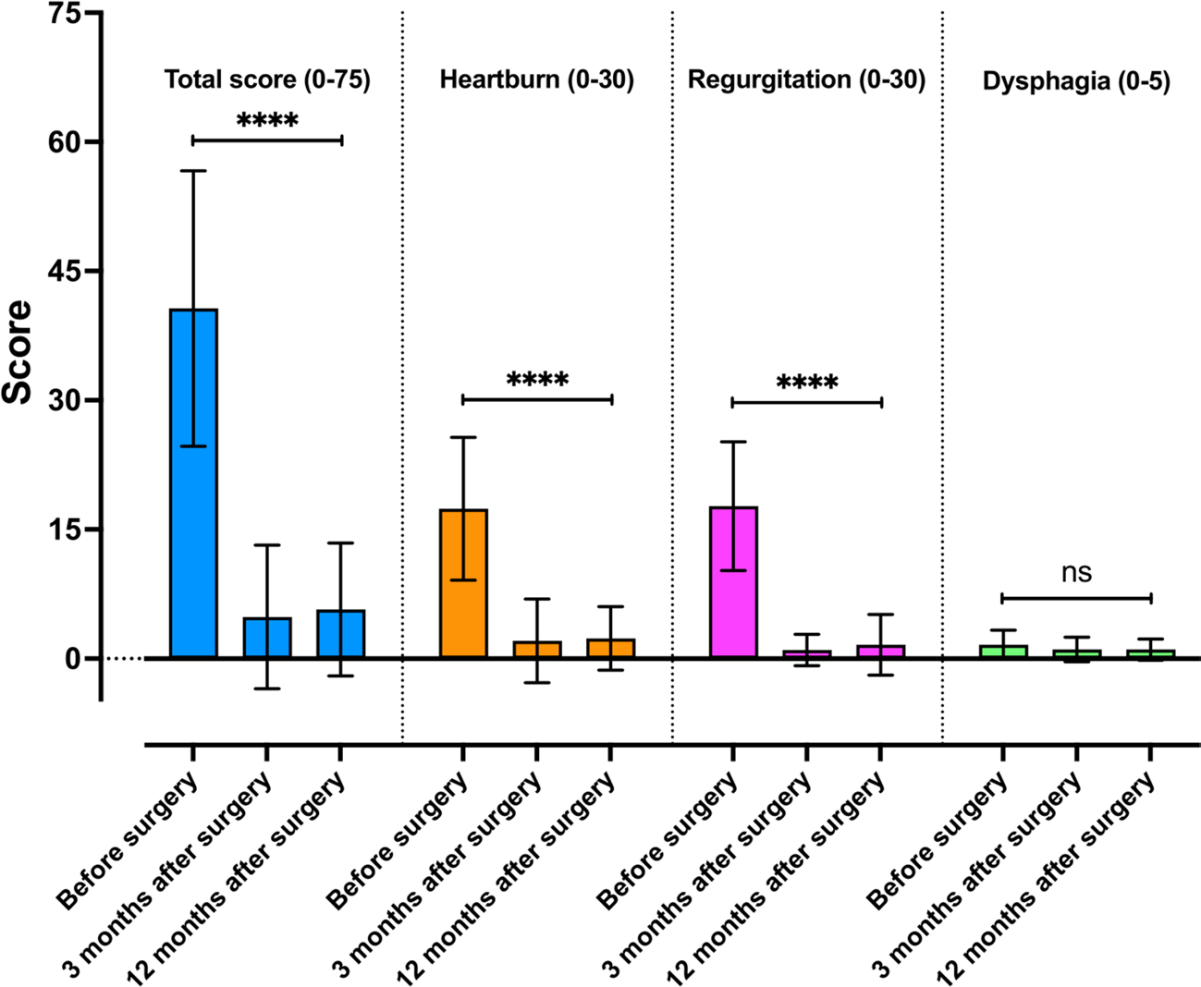
GERD-HRQL scores (0–75 points) before and 3 and 12 months after laparoscopic hiatal hernia repair with RefluxStop. Sub-scores for heartburn (0–30 points), regurgitation (0–30 points), and dysphagia (0–5 points) are presented individually. Scores are graphed as mean (box) with standard deviation (whiskers). The level of significance was set at 0.05

Based on the results of the GERD-HRQL questionnaire, all patients had improvement or complete resolution of GERD symptoms (Fig. 3).

The mean GERD-HRQL score preoperatively was 40.7 ± 16.0 and reduced to 4.8 ± 8.3 at 3 months (*p* < 0.001) and 5.7 ± 7.7 at 12 months (*p* < 0.001). Sub-scores (0–30 points) for heartburn (17.4 ± 8.3 preoperative *vs.* 2.4 ± 3.7 at 12 months) and regurgitation (17.7 ± 7.5 preoperative *vs.* 1.6 ± 3.5 at 12 months) improved after surgery in all patients (*p* < 0.001). Worsening of GERD symptoms after surgery was not reported in any of the patients at 12 months. Ninety percent of the patients reported being satisfied with their current quality of life related to GERD compared to 25% before surgery. Sixteen patients (80%) were able to completely discontinue their therapy with proton pump inhibitors.

Based on GERD-HRQL scores, results were observed to be excellent in 13 patients (65%), good in two (10%) patients, fair in three (15%) patients, and poor in two (10%) patients at the 12-month visit. Among the two patients with poor results, one patient presented with recurrence of GERD symptoms after 1 year. More detailed discussion with the patient found that he had gained 10 kg in weight (attributed to changes in his lifestyle and social environment). At the 6-month follow-up, the patient had been asymptomatic, and he reported recurrence of symptoms after 9 months. Endoscopy at 12 months postoperatively showed no signs of recurrence of reflux, and a competent cardia, with the RefluxStop device in place. However, recurrence of reflux was observed on a 48-h pH study with the Bravo capsule (DeMeester score of 60.0). The other patient had persistence of symptoms, especially heartburn, for which no cause could be identified on objective testing, and was considered multifactorial in nature.

**Fig. 4:**
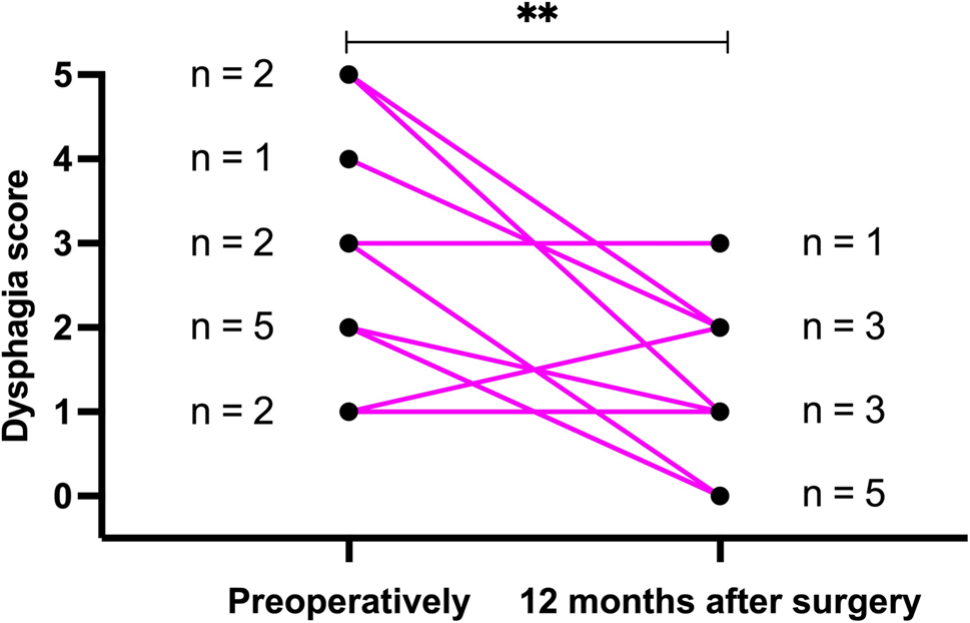
Individual evolution of the severity of dysphagia (0–5 points) 12 months after surgery in the 12 patients presenting preoperatively with dysphagia

The preexisting dysphagia encountered in 12 patients was significantly improved after surgery with a mean score of 2.7 ± 1.4 at baseline compared with 1.0 ± 1.0 after 12 months (*p* < 0.005). Individual changes over time are shown in Fig. 4. Among the 8 patients without preoperative dysphagia, 5 patients did not develop dysphagia, and 3 patients described dysphagia after 12 months. The mean score for dysphagia was 0 ± 0 at baseline compared with 1.1 ± 1.6 at 12 months (*p* < 0.075) in these 8 patients.

## Discussion

This is the first study describing experience with the RefluxStop device specifically in the treatment of patients with GERD and HH with concurrent IEM, providing data on the feasibility and safety of the RefluxStop device in this cohort of 20 patients. The results indicate that surgery with RefluxStop was feasible and safe in this patient group. Notably, we found all patients with IEM had improvement or complete resolution of GERD symptoms post-RefluxStop implantation, and 90% of the patients reported treatment satisfaction. No device-related complications were observed in this patient cohort, highlighting the safety of the device. Although one patient had device “migration,” we suggest caution in how this event is interpreted. Scrutiny of the case led to the conclusion that the device probably moved along the channel where the deployment tool was used, due to insufficient surgical closure of this passageway, rather than migration in the sense of pressure-induced tissue damage leading to subsequent movement. It is for this reason that it is categorized as a procedural complication rather than a device malfunction. To minimize the chance of similar incidents, we would suggest a critical view of the deployment tool channel and meticulous closure.

The findings of this study are pertinent to the management of patients with GERD and IEM, since existing medical and surgical treatment options are limited in this patient group. Clinical outcomes with LARS are considered more reliable or predictable in patients with normal esophageal motility, good response to proton pump inhibitors, and typical symptoms [12], meaning that patients outside of these groups, such as those with IEM and/or preoperative dysphagia, are often treated conservatively. In cases where surgery is performed, the most common approach has been to tailor the surgical technique based on esophageal motility, performing a Nissen fundoplication (full wrap) in patients with normal motility and a Toupet (partial or 270-degree wrap) in those with dysmotility [6, 13]. There are also centers that favor the Dor fundoplication (anterior wrap) in these patients, in an attempt to keep postoperative dysphagia rate low [14]. In this context, the RefluxStop device is a promising new addition in the management of patients with GERD and IEM. The general safety and effectiveness of the RefluxStop device in treating GERD has previously been demonstrated in a prospective, single-arm, multicenter study that showed it was successful in improving both GERD-HRQL score (by 86%) and 24-h pH outcomes (in 98% patients) at 1-year follow-up [3]. Although the sample size in the present study is relatively small, we would hope that these initial findings highlight the option of this surgical treatment to those involved in the care of this type of patient. By way of comparison from the literature—though this is not a control group—Addo et al. found, in a single-institution, retrospective review of a LARS (mostly Nissen, some Toupet) patient database, that satisfaction between IEM and non-IEM patients was similar (80% in IEM vs. 77.9% in non-IEM), but that those with IEM were more likely to not see an improvement in dysphagia, with dysphagia rates decreasing from 59.1 to 42.9% in the IEM group, compared to 57.2 to 31.9% in the non-IEM group) [15].

A recent publication by the American Forget Society described the multiple components that contribute to the anti-reflux barrier, most notably the crural diaphragm, the LES and its sling fibers, and an intact gastro-esophageal flap valve for which the importance of intra-abdominal esophagus length and an acute angle of His are acknowledged [16]. RefluxStop surgery aims to address all these components. In the procedure, in order to reduce the HH and achieve sufficient esophageal length to position the LES in the abdominal cavity, the distal esophagus is first dissected in the mediastinum—an abdominal length of 4–4.5 cm is required to set up the correct plication and implant positioning. Second, the angle of His and the flap valve are recreated via a gastro-esophageal plication of 90–110 degrees as a second barrier to reflux. Third, the position of the RefluxStop implant 1–1.5 cm above the LES in a fundic pocket stabilizes the LES in the abdominal cavity; thus, slippage or herniation of the LES is prevented, unless through a large defect in the diaphragm.

To create a neo-valve below the hiatal closure, most of the reflux procedures until 2018 widely adopted the principle of encircling fully or partially the distal esophagus. The RefluxStop procedure is therefore the only surgical technique that, without full or partial encircling, positions the LES well below the diaphragm. Unlike other devices, such as the LINX™ or the Angelchick procedures, the RefluxStop device is not placed circumferentially, nor does it compress or inflate/fill like a gastric band, which can cause pressure and ischemia and lead to tissue damage and ultimately migration or perforation of the device.

Some previous studies on wrap techniques have observed herniation of the wraps (e.g., telescope-herniation) despite several additional stitches. The “soft” condition of a wrap also makes posterior slippage possible due to a small recurrence of the hiatal surface area opening, allowing recurrence of the HH and GERD symptoms, often leading to dysphagia, nausea, or recurrent vomiting [17]. The RefluxStop device aims to “stabilize” the stomach fundus, blocking such slippage or recurrence of HH. In the present study, no patients had recurrence of HH in the 12-month follow-up period, though longer-term follow-up is crucial to confirm this fact. Despite 60% of the patients in the study having preoperative dysphagia, the postoperative transient dysphagia was acceptable: endoscopic balloon dilation for severe persistent dysphagia was performed in 3 patients (of whom one had new-onset dysphagia). Since the RefluxStop technique forms only a 90–110 degree plication, it allows the esophagus to distend freely to let the food bolus pass. Nevertheless, HH repair or technical failure while suturing the plication could still lead to dysphagia. Placing too much tension on the two vertical running sutures while forming the esophago-gastric plication could potentially create kinking or narrowing of the esophagus, causing an amotile or weak area. Therefore, surgical expertise including advanced suturing skills is necessary to achieve accurate positioning of the RefluxStop implant. Based on our own clinical experience, we would suggest that any experienced and skilled foregut surgeon performing at least 20–25 Nissen or Toupet fundoplications a year should reasonably be able to position the device, given a learning curve of around 5–10 cases.

In this procedure, the extensive dissection of the esophagus and repositioning of the LES into the abdomen, and securing its position with the RefluxStop device, will lead to straightening of the esophagus and recreation of tension, which could then encourage/facilitate emptying of the esophagus—we postulate that this could benefit patients with weak or ineffective motility and a HH.

This study has some limitations. There is no control group, leading to a selection bias as results presented were only from patients with IEM who underwent the RefluxStop procedure. The follow-up period of 12 months may be too short to assess longevity of outcomes and recurrence of HH and other complications, such as implant migration and/or penetration. However, to date, only one published study has reported data about surgery with RefluxStop, and as this is a new technique, we consider it reasonable to present our data on the first 20 patients suffering from GERD with concurrent IEM operated with the RefluxStop device at this time point. Future studies are underway to directly compare patients with and without IEM in a larger pooled cohort. In the present study, esophageal motility was largely determined on the basis of video-esophagram, with manometry performed in selected cases when video-esophagram results were equivocal. High-resolution manometry is generally considered the gold standard method of testing [4, 18, 19], due to the lower sensitivity of video-esophagram, though it is possible to identify more obvious motility disorders on video-esophagram [20, 21]. If video-esophagram is abnormal in a patient with preoperative dysphagia, it is very unlikely that the motility study would have different findings, if performed correctly in supine and upright position.

## Conclusion

The findings of this study in a cohort of 20 patients with IEM indicate the suitability of surgery with RefluxStop in this patient group, based on the feasibility, safety (no device-related peri- or postoperative complications), and effectiveness in reducing symptoms. Postoperative dysphagia was prominent in three patients and could be resolved in all patients after serial dilations. All patients who had existing dysphagia preoperatively demonstrated a reduction in intensity of symptoms or complete resolution at 3-month follow-up. Overall, all patients described an improvement, and 90% of them were satisfied with their quality of life 1 year after surgery. Long-term clinical studies with larger patient population and a control group will be beneficial to strengthen the evidence for the use of RefluxStop to treat patients with GERD and IEM.

## Data Availability

Data available on request after IRB approval.
